# Determinants of accelerated metabolomic and epigenetic aging in a UK cohort

**DOI:** 10.1111/acel.13149

**Published:** 2020-05-03

**Authors:** Oliver Robinson, Marc Chadeau Hyam, Ibrahim Karaman, Rui Climaco Pinto, Mika Ala-Korpela, Evangelos Handakas, Giovanni Fiorito, He Gao, Andy Heard, Marjo‐Riitta Jarvelin, Matthew Lewis, Raha Pazoki, Silvia Polidoro, Ioanna Tzoulaki, Matthias Wielscher, Paul Elliott, Paolo Vineis

**Affiliations:** ^1^ MRC Centre for Environment and Health Department of Epidemiology and Biostatistics School of Public Health Imperial College London London UK; ^2^ Computational Medicine, Faculty of Medicine University of Oulu and Biocenter Oulu Oulu Finland; ^3^ NMR Metabolomics Laboratory School of Pharmacy, University of Eastern Finland Kuopio Finland; ^4^ Laboratory of Biostatistics Department of Biomedical Sciences University of Sassari Sassari Italy; ^5^ Italian Institute for Genomic Medicine (IIGM, former HuGeF) Candiolo Italy; ^6^ Center for Life Course Health Research Faculty of Medicine University of Oulu and Unit of Primary Health Care Oulu University Hospital Oulu Finland; ^7^ Department of Life Sciences College of Health and Life Sciences Brunel University London Uxbridge UK; ^8^ National Phenome Centre Department of Metabolism Digestion and Reproduction Imperial College London London UK

**Keywords:** accelerated aging, affective mood disorders, DNA methylation, metabolomics, molecular biology of aging, risk factors

## Abstract

Markers of biological aging have potential utility in primary care and public health. We developed a model of age based on untargeted metabolic profiling across multiple platforms, including nuclear magnetic resonance spectroscopy and liquid chromatography–mass spectrometry in urine and serum, within a large sample (*N* = 2,239) from the UK Airwave cohort. We validated a subset of model predictors in a Finnish cohort including repeat measurements from 2,144 individuals. We investigated the determinants of accelerated aging, including lifestyle and psychological risk factors for premature mortality. The metabolomic age model was well correlated with chronological age (mean *r* = .86 across independent test sets). Increased metabolomic age acceleration (mAA) was associated after false discovery rate (FDR) correction with overweight/obesity, diabetes, heavy alcohol use and depression. DNA methylation age acceleration measures were uncorrelated with mAA. Increased DNA methylation phenotypic age acceleration (*N* = 1,110) was associated after FDR correction with heavy alcohol use, hypertension and low income. In conclusion, metabolomics is a promising approach for the assessment of biological age and appears complementary to established epigenetic clocks.

## Graphical abstract

We have developed a novel assessment of biological age using broad metabolomic profiling of small molecules circulating in blood and urine, collected from over 2,000 working age adults. We compare this metabolomic age assessment with an established epigenetic ‘clocks’ and show that ‘age acceleration’ is associated with risk factors of premature mortality, including depression.

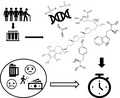

## 1 INTRODUCTION

Aging has been defined as the “time‐dependent decline of functional capacity and stress resistance, associated with increased risk of morbidity and mortality” (Burkle et al., 2015). Environmental stressors, including lifestyle and social adversity (Stringhini et al., 2018), psychological disorders (Chiu et al., 2018; Wolf & Morrison, 2017), and genetic factors (McDaid et al., 2017) may influence the aging process, leading to differing aging rates. Traditionally, quantitative assessment of “the rate of aging” relies on the analysis of mortality curves of populations. However, at the level of a single individual, this method does not allow assessment of the state of aging (i.e., the state of the functional decline) and a prediction of the risk of morbidity and remaining life expectancy. In contrast, markers of “biological age” that can be assessed at any point in the lifespan may allow, for instance, early identification of individuals or groups at risk of developing age‐related disease or frailty and enable targeted interventions (Ferrucci, Levine, Kuo, & Simonsick, 2018).

Since aging is a process that affects almost all tissues and organs and involves crosstalk between multiple physiological systems, there has been increased research into composite markers of aging, involving multiple parameters (Jylhävä, Pedersen, & Hägg, 2017). Biological age scores have been developed by combining established clinical biomarkers (Levine, 2013) (Belsky et al., 2015) and have been associated with measures of functional decline such as cognitive ability (Belsky et al., 2015). Modern “omics” platforms have provided new opportunities for the systematic and agnostic assessment of biological aging. Analysis of genome‐wide DNA methylation (Hannum et al., 2013; Horvath, 2013; Levine et al., 2018), mRNA (Peters et al., 2015) and miRNAs (Huan et al., 2018) has allowed the development of multi‐parameter “omic clocks,” built upon molecular changes that tick at an average rate consistent with chronological age. DNA methylation age acceleration, defined as having a greater DNA methylation age than chronological age (i.e., a faster than average “ticking rate”), is associated with multiple noncommunicable disease (NCD) risk factors (Fiorito et al., 2019) and predictive of aging outcomes such as frailty, cognitive decline (Horvath et al., 2016) and all‐cause mortality (Chen et al., 2016; Dugue et al., 2018).

Metabolomics, the profiling of small molecules, is a promising technology for the comprehensive assessment of biological aging. As the final product of cellular metabolism, metabolites may provide a more complete picture of biological processes and a stronger phenotypic representation than other “omic profiles.” Although metabolomic studies have reported strong associations between metabolites and age, these have been of limited sample size (Chaleckis, Murakami, Takada, Kondoh, & Yanagida, 2016; Rist et al., 2017) or employed targeted analyses with only partial coverage of the full metabolome (Auro et al., 2014) (Hertel et al., 2016; Yu et al., 2012). Larger sample sizes can provide more precise, and potentially less biased, assessments of a typical biological age in a given population, while the use of multiple analytical platforms can provide a more complete assessment of metabolic processes relevant to biological aging. Only one study provided an overall assessment of biological aging, based on urinary proton nuclear magnetic resonance spectroscopy (NMR), reporting that metabolomic age was associated with time to death, after adjustment for chronological age and other risk factors (Hertel et al., 2016).

In the present study, we aimed to develop a systematic assessment of biological age using untargeted metabolomics. We have employed multiple metabolomic analytical platforms, providing unprecedented metabolome coverage, to develop a predictive model of age, within a large sample from the Airwave cohort of employees of the police service in Great Britain. A second cohort was used for longitudinal validation of selected metabolic age predictors. To assess whether deviations between the predicted metabolomic age and chronological age reflect differences in biological aging rate, we have investigated associations with risk factors of premature mortality, including the WHO “25 × 25” risk factors (World Health Organisation, 2013) (hypertension, diabetes, obesity, smoking, alcohol use and physical inactivity) and socio‐economic and psychological risk factors (income, depression, anxiety, post‐traumatic stress disorder (PTSD)). Finally, to assess whether metabolomic age is complementary to established epigenetic assessments of age, we have assessed the relationship between metabolomic and epigenetic aging, and their relative associations with risk factors.

## 2 RESULTS

### 2.1 Study population

The study population comprised 2,238 participants of the Airwave cohort with full metabolomic data. A 60.5% of participants were male, and mean age was 41.24 years (*SD*: 9.1, range: 19.2–65.2 years). Most participants (97.5%) were of white British ethnicity, and 27.8% of participants were educated to degree level. The demographic characteristics of this sample are representative of the wider cohort (Elliott et al., 2014).

### 2.2 Metabolomic age modelling

Metabolomic data were acquired from both urine and serum samples using multiple proton nuclear magnetic resonance spectroscopy (NMR) and ultra‐performance liquid chromatography–mass spectrometry (UPLC‐MS) platforms, providing in total nine different metabolomic data types (Table 1).

**TABLE 1:** Summary of metabolomic platforms used in analysis, including predictive performance in single platform analysis

Platform	Abbreviation	Details	*N* data points^a^	*r* ^b^	*MAE* ^b^
MS HPOS in serum	sHPOS	Hydrophilic interaction chromatography (HILIC), provides enhanced separation of small, highly polar molecules ionized in positive mode	1,505	.62 (.56, .67)	5.69 (5.34, 6.07)
MS LNEG in serum	sLNEG	Lipid‐targeted reversed‐phase chromatography provides maximal resolution of fatty acids, triglycerides, and phospholipids, ionized in negative mode	5,833	.71 (.67, .75)	5.07 (4.76, 5.37)
MS LPOS in serum	sLPOS	Lipid‐targeted reversed‐phase chromatography, ionized in positive mode	7,211	.80 (.77, .83)	4.29 (4.06, 4.55)
NMR BiLISA in serum	sBiLISA	Quantifies cholesterol, free cholesterol, phospholipids, triglycerides, apolipoprotein A1, A2, B and particle numbers for the primary lipoproteins and their subclasses	105	.45 (.39, .51)	6.49 (6.19, 6.79)
NMR CPMG in serum	sNMR	Highly robust, repeatable and precise platform	23,571	.65 (.62, .69)	5.53 (5.21, 5.82)
MS HPOS in urine	uHPOS	Hydrophilic interaction chromatography (HILIC), provides enhanced separation of small, highly polar molecules ionized in positive mode	7,325	.77 (.74, .80)	4.62 (4.27, 4.95)
MS RNEG in urine	uRNEG	Reversed‐phase chromatography targets small moderately polar molecules, ionized in negative mode	14,481	.79 (.75, .82)	4.42 (4.14, 4.70)
MS RPOS in urine	uRPOS	Reversed‐phase chromatography, ionized in positive mode	14,300	.83 (.80, .85)	4.17 (3.92, 4.38)
NMR NOESY in urine	uNMR	Highly robust, repeatable and precise platform	24,493	.58 (.52, .62)	5.88 (5.62, 6.20)

^a^ Refers to spectral data points in uNMR and sNMR, to lipoprotein and subclass measures in sBiLISA and retention time‐m/z pairs in MS analysis.

^b^ *r* and *MAE* refer to the mean Pearson's correlation and mean absolute error, respectively, with chronological age across the independent test sets, with brackets showing bootstrapped 95% confidence internals.

#### 2.2.1 Age prediction by platform

We first assessed the age prediction of each metabolomic platform separately (Table 1). We followed a bootstrapping procedure and trained elastic net models of age in re‐sampled training portions (80%) of our data 100 times, for each metabolomic platform. Predictive performance was then assessed in the independent training sets (remaining 20% of data) to provide unbiased estimates. The best performing platforms were reversed‐phase UPLC‐MS in positive mode in urine (“uRPOS”) and lipid‐targeted reversed‐phase UPLC‐MS in positive mode in serum (“sLPOS”) that had mean correlations between predicted and chronological age of 0.83 (bootstrap 95% confidence interval (CI): 0.80, 0.85) and 0.80 (95% CI 0.77, 0.82), respectively, across testing sets. The worst performing platforms were NMR in urine (NOESY experiment, “uNMR”) and Bruker IVDr Lipoprotein Subclass Analysis derived from NMR in serum (“sBiLISA”) with mean correlations between predicted and chronological age of 0.58 (CI: 0.52, 0.62) and 0.45 (95% CI 0.39,0.51), respectively, across testing sets.

#### 2.2.2 Multi‐platform metabolomic age

To find the combination of metabolomic data sets that provided the best age prediction performance, we combined the metabolomic data sets and constructed further elastic net models of age, sequentially leaving one platform out each time. Predictive performance of age (minimization of mean squared error (*MSE*) in 10‐fold cross‐validation was improved by use of only the four following platforms (Figure S1): sBiLISA, sLPOS, uRPOS and hydrophilic interaction UPLC‐MS in positive mode in urine (“uHPOS”).

**FIGURE 1:**
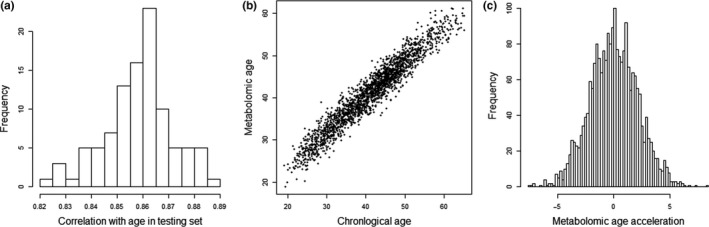
Summary of metabolomic age prediction. (a) Distribution of Pearson's correlation coefficient (*r*) between chronological and predicted age across bootstrapped test sets. (b) Metabolomic age plotted against chronological age. (c) Distribution of metabolomic age acceleration scores

These four platforms were retained to give a total of 28,941 metabolic features (retention time‐mz pairs or lipoprotein measurements) for the final stage of model building. To define a stable metabolomic age score for each participant, we again followed the bootstrapping procedure of re‐sampling training and testing portions of this data set 100 times. Prediction of age in the independent training sets was highly accurate and stable across all re‐samplings with mean correlation between predicted and chronological age of 0.86 (95% CI: 0.85, 0.88) (Figure 1a). Mean absolute error (MAE) across training sets was on average 3.71 years, with a bootstrap 95% CI of 3.37 to 3.96 years. The metabolomic age of each participant was assigned as their mean predicted age across the 100 bootstrapped models (Figure 1b). Metabolomic age acceleration (mAA) was then defined as the difference, at a given age, between chronological and metabolomic age (Figure 1c).

**FIGURE 2:**
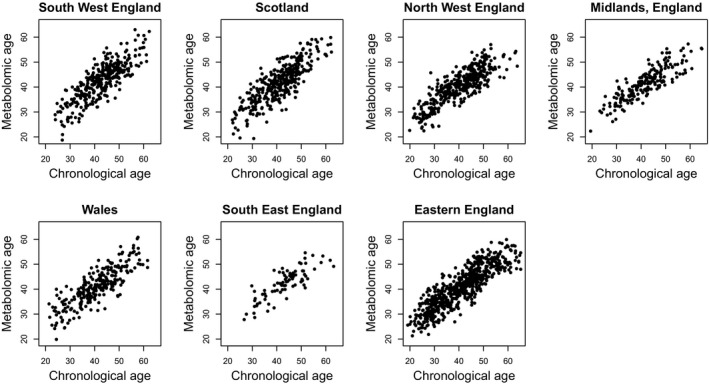
Metabolomic age plotted against chronological age across different independent study areas. Seven models in this analysis were trained separately on data from participants in six out of the seven study areas and validated with data in the remaining study area shown

Age prediction was also stable across different population areas (Table S1, Figure 2). Correlation between predicted and chronological age across different police services of Great Britain (using remaining study areas as training sets to predict age in each respective study area) ranged from 0.82 in Scotland to 0.87 in the North West of England.

### 2.3 Metabolomic age predictors

The median number of metabolic features selected into each model in the final stage was 1,311 (range: 498–9,754) (see Data Set S1 for list of predictors along with Data Set S2 annotation information). 62 metabolic features were present in 100% of models, 450 metabolic features were present in at least 75%, 1,080 metabolic features were present in at least 50%, and 2,990 were present in only one model. Features present in at least 75% included 8 lipoprotein subclasses from sBiLISA and 207, 81 and 154 features (retention time‐m/z pairs) from the sLPOS, uHPOS and uRPOS platforms, respectively.

**FIGURE 3:**
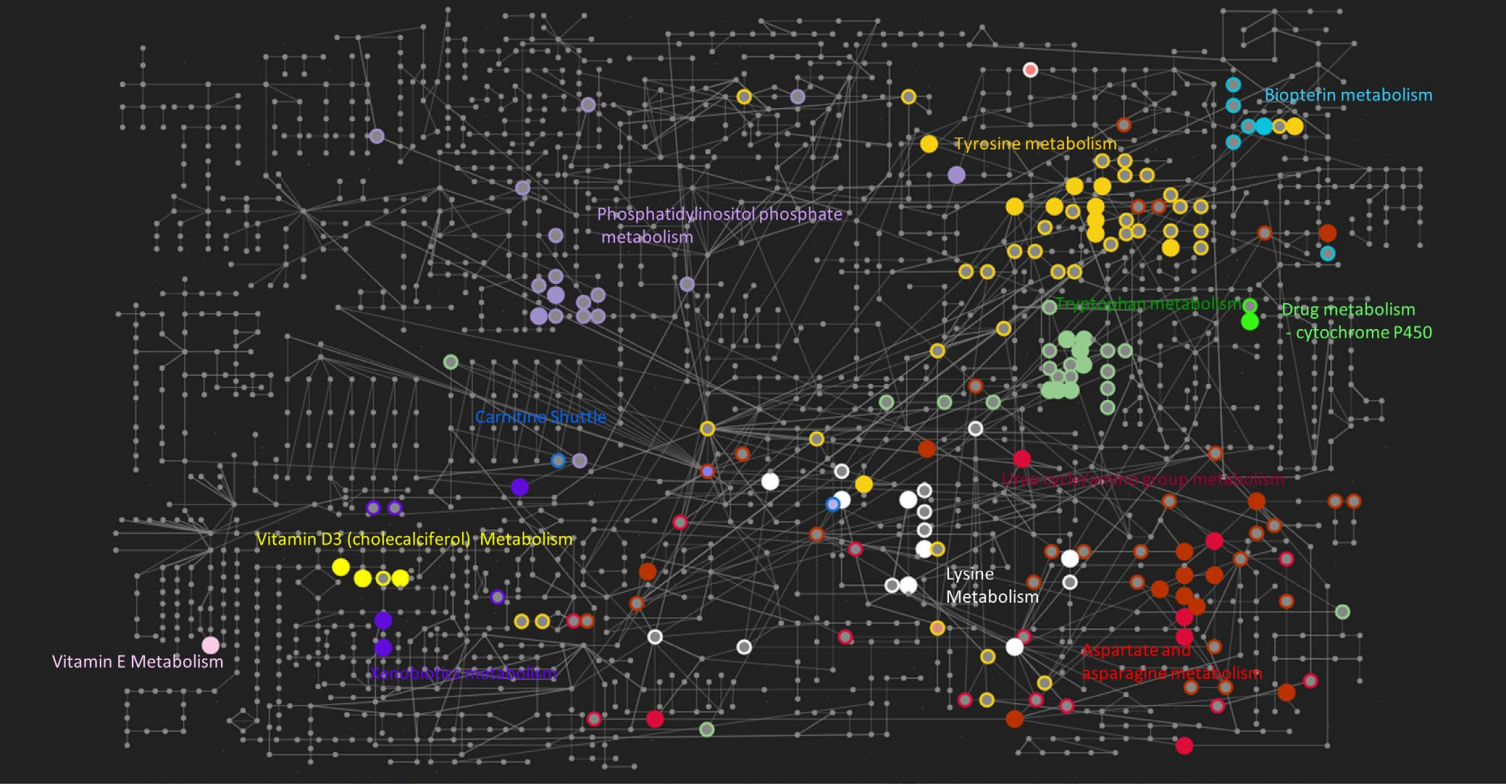
Metabolic network visualisation of significantly enriched pathways based on the manually curated KEGG global metabolic network (Chong et al., 2018). The metabolites of significantly enriched pathways are represented as nodes on the network. Empty nodes represent compounds identified from the feature list by *Mummichog* but not significant, while solid nodes represent significantly enriched features. Note not all metabolites from the KEGG global network are displayed

Pathway enrichment analysis, using the *Mummichog* algorithm performed across the UPLC‐MS‐derived features that were present in at least 75% of models identified enrichment in thirteen metabolic pathways (Table 2, Figure 3): vitamin E metabolism; lysine metabolism; urea cycle/amino group metabolism; vitamin D_3_ (cholecalciferol) metabolism; tryptophan metabolism; carnitine shuttle; phosphatidylinositol phosphate metabolism; aspartate and asparagine metabolism; drug metabolism—cytochrome P450; biopterin metabolism; xenobiotic metabolism; butanoate metabolism; and tyrosine metabolism.

**TABLE 2:** Significantly enriched metabolic pathways among metabolomic age predictors present in at least 75% of models

Pathways	Overlap size^a^	Pathway size^b^	*p*‐value^c^
Vitamin E metabolism	16	37	.0129
Lysine metabolism	11	29	.0161
Urea cycle/amino group metabolism	17	52	.0170
Vitamin D_3_ (cholecalciferol) metabolism	5	10	.0185
Tryptophan metabolism	20	69	.0218
Carnitine shuttle	11	34	.0219
Phosphatidylinositol phosphate metabolism	9	27	.0231
Aspartate and asparagine metabolism	20	71	.0241
Drug metabolism—cytochrome P450	15	52	.0256
Biopterin metabolism	5	15	.0354
Xenobiotic metabolism	18	71	.0397
Butanoate metabolism	7	26	.0474
Tyrosine metabolism	22	91	.0478

^a^ The number of model predictors (sLPOS, uHPOS and uRPOS) matched to each pathway.

^b^ The number of metabolites in the whole sLPOS, uHPOS and uRPOS data sets matched to each pathway.

^c^ *p* values adjusted for type 1 error through Gamma‐based permutation procedure..

We examined concentration changes of eleven metabolites included in over 50% of age prediction models, that were available in an independent cohort, the Northern Finnish Birth Cohort 1966, that had serum NMR metabolomic data measured at two ages, 31 and 46 years, among 2,144 individuals. Nine of these metabolites (82%) changed significantly with age (validation *p* < .005), in the same direction as predicted in the metabolomic age model (Table S2).

### 2.4 Comparison with DNA methylation age

DNA methylation age was assessed for 1,110 participants, using the multi‐tissue clock derived by Horvath (Horvath, 2013) and the blood‐derived clock of Hannum et al. (2013) both trained on chronological age, and the clock of Levine et al. (2018) that was trained on “phenotypic age.” Demographic characteristics for this sample were similar to those for participants with metabolomic age available (age range = 19.9–65.2 years, 60% male). DNA methylation age measures were strongly correlated with chronological age (Table 3) and metabolomic age (for 837 participants with both metabolomic and epigenetic data, Table 3). Mean age acceleration scores were 0.00 (standard deviation (*SD*): 3.65), 0.00 (*SD*: 3.19) and 0.01 (*SD*: 5.12) years for Horvath, Hannum and Phenotypic ages, respectively, while mean mAA was 0.00 years (*SD*: 2.13 years). No correlation was observed between epigenetic age acceleration measures and mAA (Table 3).

**TABLE 3:** Pearson's correlations between the various age measures

	Age (chronological)	Metaboomic age	Metabolomic AA	DNAm age (Horvath)	DNAm AA (Horvath)	DNAm age (Hannum)	DNAm AA (Hannum)	DNAm phenotypic age
Metabolomic age^a^	.96							
Metabolomic AA	−.01	.27						
DNAm age (Horvath)	.89	.87	.02					
DNAm AA (Horvath)	0	.01	.04	.45				
DNAm age (Hannum)	.92	.88	−.01	.9	.17			
DNAm AA (Hannum)	0	−.01	−.01	.19	.43	.39		
DNAm phenotypic age	.86	.83	0	.83	.15	.87	.19	
DNAm phenotypic AA	.01	.02	.02	.16	.32	.17	.37	.5

Abbreviation: AA, age acceleration.

^a^ Refers to mean predicted age across all bootstrapped metabolomic models.

### 2.5 Risk factors of age acceleration

**FIGURE 4:**
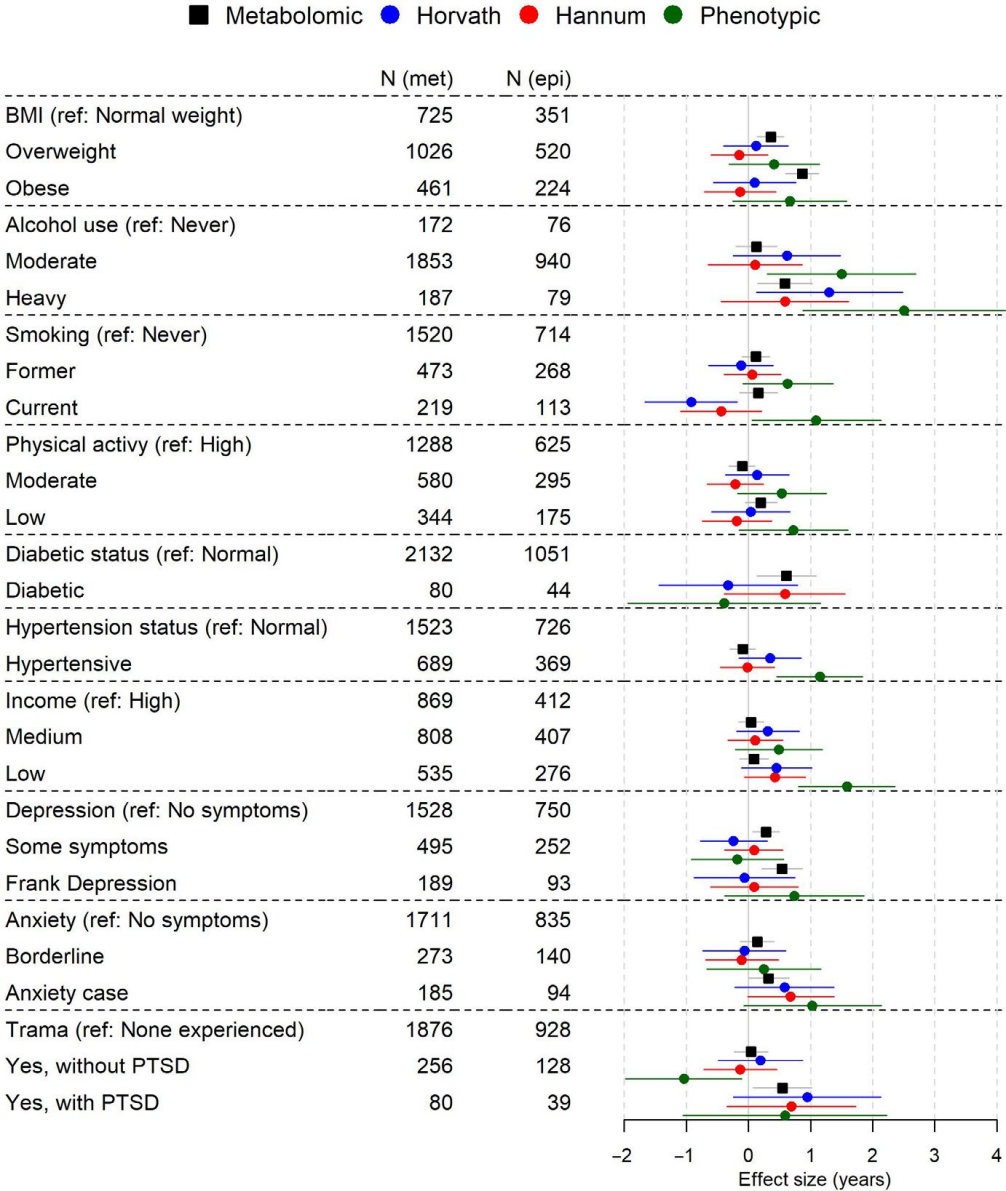
Associations between risk factors of premature mortality and age acceleration scores. Models adjusted for sex, ethnicity, study centre, income, hypertension, diabetes, BMI, smoking, alcohol intake, physical activity, and fruit, vegetable, meat and fish consumption. Bars show 95% confidence intervals. *N* (met) and *N* (epi) columns indicate number analysed for each category for metabolomic age and DNA methylation age measures, respectively

Figure 4 and Table S3 shows adjusted associations with age acceleration measures for noncommunicable disease and psychological risk factors (adjusted for sex, ethnicity, study centre, income, hypertension, diabetes, body mass index (BMI), smoking, alcohol intake, physical activity, and fruit, vegetable, meat and fish consumption). We observed increases (*p* < .05) in mAA with overweight, obesity, heavy drinking, diabetes, depressive symptoms, depression, anxiety and post‐traumatic stress disorder ranging from 0.32 (interpretable as years of age acceleration, 95% confidence interval (CI): 0.00, 0.65) for anxiety compared to those without any symptoms of anxiety, to 0.87 (95% CI: 0.60, 1.13) for obesity (BMI ≥ 30 kg/m^2^) compared to those of normal weight (BMI < 25 kg/m^2^). Associations of mAA with overweight, obesity, heavy drinking, diabetes, depressive symptoms and depression remained significant after correction for false discovery rate.

Increases with DNA methylation age acceleration were observed for low income (Hannum: 0.43, 95% CI: −0.06, 0.92), anxiety (Hannum, 0.68, 95% CI: −0.01, 1.38) and PTSD (Horvath: 0.95, 95% CI: −0.24, 2.13) that were of a larger size than for mAA. Effect sizes with phenotypic age acceleration were generally larger than for the other age acceleration measures and in the expected direction for all risk factors, except for diabetes. In particular, associations between phenotypic age acceleration with heavy alcohol use (2.51, 95% CI: 0.88, 4.13), hypertension (1.09, 95% CI: 0.06, 2.13) and low income (1.59, 95% CI: 0.81, 2.36) remained significant after correction for false discovery rate.

### 2.6 Sensitivity analysis

To avoid the possibility of metabolic features being selected into models due to their association with body mass index (BMI) (that increases with chronological age and is associated with widespread disturbances in the metabolome (Elliott et al., 2015)), we repeated the metabolomic age modelling process, with BMI of participants included as a predictor, forced into all models. Similar results were obtained (mean correlation across test set = 0.86, bootstrap 95% CI: 0.84, 0.88). Enriched metabolic pathways among these models were similar (Table S4). mAA was calculated from these models as before but additionally adjusting for BMI to remove its contribution from the mAA scores. Similar associations with the new BMI‐adjusted mAA score and risk factors were observed (Table S5).

## 3 DISCUSSION

We have demonstrated in a large nationwide cohort study that metabolomic profiling may be used to predict chronological age with high accuracy among working‐age adults. We employed a wide range of metabolomic platforms to provide the broadest metabolome coverage yet presented in population‐based studies. We found that metabolomic age acceleration, defined as having a greater predicted metabolomic age than chronological age, was associated with overweight/obesity, high alcohol intake, diabetes and depression. We did not observe an association between epigenetic age acceleration and metabolomic age acceleration, suggesting these measures capture separate aspects of the aging process. We observed larger effect sizes of epigenetic than metabolomic age acceleration with anxiety‐related disorders.

The correlation between chronological and our measure of metabolomic age (mean *r* = .86 in the validation data sets) was somewhat lower than that of the Hannum epigenetic age clock in our cohort (*r* = .92) but greater than reported for other biological aging markers, including the measure based on urinary NMR data (Hertel et al., 2016) (*r* = .53 in men and 0.61 in women in validation data set), the blood transcriptomic clock (Peters et al., 2015) (*r* = .35–.74 depending on cohort) and telomere length (*r* ~ .3 (Muezzinler, Zaineddin, & Brenner, 2013)). Biological aging markers aim to better capture the body's rate of decline or physiological breakdown than chronological age itself and should therefore also be more predictive of mortality and age‐related disease. The associations, we observed between accelerated metabolomic aging and factors known to increase risk of mortality, suggest that metabolomic age may capture this physiological decline.

Strong direct associations with mAA were observed with diabetes, overweight and obesity. These conditions are forms of metabolic dysregulation, and their additional metabolic burden may increase the rate of decline of the metabolic systems of the body. Genetic predisposition to longevity is associated with low levels of abdominal visceral fat (Sala et al., 2015) and many different conditions that prolong lifespan in animal models also improve obesity‐related conditions. Furthermore, obesity has been linked to telomere shortening, and drastic measures to combat morbid obesity like bariatric surgery can cause a recovery in telomere length (Laimer et al., 2015). Much is now known about the aging process at the molecular level primarily from experimental work. López‐Otín, Blasco, Partridge, Serrano, and Kroemer, (2013) proposed nine “hallmarks of aging” that may be expected to have detectable effects on the metabolome and overlap significantly with the effects of metabolic disorders (López‐Otín, Galluzzi, Freije, Madeo, & Kroemer, 2016). For instance, the hallmark “deregulated nutrient signalling” refers to pathways that sense and respond to nutrient availability such as “insulin and IGF1 signalling” (IIS) pathway, which is altered in diabetes.

We observed higher mAA for individuals with depressive symptoms and those with frank depression. Both psychological distress and major depression were reported to have similar hazard ratios for mortality in a recent prospective study (Chiu et al., 2018). Consistent evidence demonstrates a bi‐directional association between depression and so‐called metabolic syndrome, suggesting common pathological roots (Marazziti, Rutigliano, Baroni, Landi, & Dell'Osso, 2014). Proposed pathophysiological commonalities include abnormal activation of the hypothalamic–pituitary–adrenal (HPA) axis and altered levels of circulating leptin and ghrelin, two peripheral hormones that are classically implicated in the homeostatic control of food intake. While in this cross‐sectional study we cannot disentangle the causal direction between depression and mAA, a study of biological aging among elderly people found that accelerated biological age was associated with depressive symptoms at baseline and was also predictive of depressive symptoms at follow‐up (Brown et al., 2017).

In pathway analysis of metabolomic age model predictors, we observed enrichment of the tryptophan, tyrosine and biopterin metabolic pathways that relate to the aging hallmark “altered intercellular communication.” Tyrosine is required for signal transduction through incorporation into protein kinases, while tryptophan and biopterin are necessary for synthesis of neurotransmitters including dopamine, norepinephrine, epinephrine, serotonin and melatonin. Alterations to neurotransmitter levels may underlie the associations we observed between mAA with depressive symptoms and depression. Enrichment of pathways including lysine metabolism, carnitine shuttle, urea cycle, and aspartate and asparagine metabolism directly relate to mitochondrial dysfunction, which has long been recognized as an aging hallmark (López‐Otín et al., 2013). Dysfunctional mitochondria are major sources of genotoxic reactive oxygen species (López‐Otín et al., 2013), which impacts other aging hallmarks including genomic instability, altered intercellular communication and stem cell exhaustion (Ito et al., 2006). We observed enrichment of metabolism of vitamin E, a potent antioxidant and anti‐inflammatory agent that protects cell membranes from oxidative damage that can induce genome instability (Claycombe & Meydani, 2001), and of vitamin D_3_, whose production decreases with age (Gallagher, 2013).

We observed increases in Hannum and Horvath age acceleration associated with anxiety, PTSD and low income that were generally of greater size than for mAA (although with wider confidence intervals due to the smaller sample size). Meta‐analyses have shown that both PTSD (Wolf & Morrison, 2017) and low socio‐economic position (Fiorito et al., 2017) are associated with higher Hannum age acceleration. We did not observe any evidence for an association between depression and Hannum or Horvath age acceleration, suggesting epigenetic and metabolomic aging measures may be sensitive to separate dimensions of mental health. The DNA methylation clocks of Horvath and Hannum have been shown to perform well as markers of biological age since they are predictive of all‐cause mortality, even after adjusting for chronological age and a variety of known risk factors, and are associated with physical measures of aging such as frailty and cognitive decline (Horvath et al., 2016). However, other biological aging markers may add value in capturing different aspects of the aging process. Peters et al. (2015) reported that transcriptomic age was only moderately correlated with DNA methylation age and the different measures were associated with different aging phenotypes. Similarly, Belsky et al. (2017) report only weak correlations between telomere length, DNA methylation age and a composite biomarker‐based measure of biological aging among young adults. While metabolomic and DNA methylation age were correlated in our study, mAA and DNAmAA were not. This is likely because the platforms measure alterations related to different aging hallmarks: Horvath proposed that his clock measured maintenance of epigenetic stability, while pathway analysis suggests that metabolomic age relates to hallmarks including nutrient signalling, mitochondrial dysregulation and intra‐cellular communication. Epigenetic age acceleration measures are predictive of cancer‐related mortality but not CVD (Dugue et al., 2018; Horvath et al., 2016) while the risk factors associated with mAA suggest it may be predictive of cardio‐metabolic‐related disease. Accelerated transcriptomic age was found to be similarly associated with CVD risk factors, although it was not related to mental health (Peters et al., 2015). Further research into biological aging may consider combining markers at different levels of biological organization to provide a more complete picture of the aging process.

We generally observed stronger associations between risk factors and phenotypic age acceleration than for the other aging measures. This is to be expected as DNA methylation phenotypic age was not trained on chronological age, as for the other measures, but trained on a composite measure of clinical markers, selected based on prediction of time to death. As a marker of biological age, rather than chronological age, this approach to training aging markers has obvious advantages in capturing the age‐associated phenotypic changes most predictive of mortality and morbidity. However, this approach will also assess to a greater extent the early effects of disease, rather than the root molecular changes that underly the normal intrinsic aging process. It has been proposed that aging should be viewed as hierarchical in nature with changes at the molecular level (the “aging hallmarks”) underlying, and preceding, changes at the phenotypic level, which in turn underly levels of function and healthspan (Ferrucci et al., 2018). While training metabolomic age on mortality, as for phenotypic age, may increase sensitivity to risk factors, the current approach of assessment of molecular changes associated with chronological age therefore has other advantages in terms of identifying the root, intrinsic processes associated with biological aging, which may precede phenotypic changes.

This study has some limitations. The data analysed from Airwave were cross‐sectional, based on a single biological sampling from participants at a wide range of ages and we cannot therefore discount the contribution of cohort effects. However, pathways that were enriched in our models were related to endogenous physiological processes related to aging, and we validated selected metabolomic age predictors that were available in an independent cohort at two timepoints (15 years apart) in the early adult life of the same individuals. We found that for most of these metabolites, changes over time were as predicted by the metabolomic age model. Our population only covered working‐age adults and it is known that biological aging becomes more variable within the elderly. Further work is required to test the performance of our modelling approach for older populations.

The use of untargeted metabolomics presents both strengths and limitations. For untargeted analyses by UPLC‐MS, there may be issues in matching features across different studies or data sets because of differences in retention time and mass accuracy in different runs of spectral acquisition. This is not the case for ^1^H NMR, however, since different features can be closely aligned across studies using appropriate preprocessing steps (Karaman et al., 2016). However, NMR is less sensitive than UPLC‐MS and different molecules may produce overlapping signals in untargeted data sets introducing noise. Indeed, we found most NMR platforms were not selected for inclusion during our final modelling process. Structural annotation of relevant metabolic features was outside the scope of the present study and may not even be possible for some predictors without current database matches. However, the aim of the study was to develop an overall assessment of metabolic age rather than identify individual metabolic features. Indeed, the nature of the variable selection method used means that equally predictive models can be built on different sets of metabolites. We used the *Mummichog* pathway analysis tool to extract information at the pathway level, as the algorithm bypasses laboratory annotation based on the assumption that misidentification will apply equally both to the feature set (metabolites included in the age prediction model) and the reference set (metabolites not selected into the model). The tool has been validated in separate data sets that have also undergone full laboratory annotation (Li et al., 2013). We incorporated a range of MS platforms able to detect both lipophilic and hydrophilic molecules at low concentrations and NMR platforms able to detect larger structures such as lipoproteins that would be destroyed during MS acquisition. We also analysed both serum and urine that contain different sets of metabolites—more lipophilic molecules in serum and more polar molecules that are present at higher concentrations in urine. Together, we assayed a large portion of the metabolome that would not be possible with current targeted methods. Other strengths include the incorporation of DNA methylation data, the wide age range of participants including those in early adult life where aging interventions may be most effective (Moffitt, Belsky, Danese, Poulton, & Caspi, 2017) and the use of validated psychological instruments.

In conclusion, we have developed a predictive indicator of aging based on broad metabolomic analysis among working‐age adults. We found that while mAA, the difference between metabolomic and chronological age, was not related to epigenetic age acceleration, it was associated with mortality risk factors including overweight/obesity, diabetes, heavy alcohol use and depression. Biological age acceleration may be an important mechanism linking affective mood disorders to age‐related disease. Advances in life expectancies have led to an increased prevalence of age‐related morbidities. Targeting the process of aging itself, through changes in living conditions, behaviours or therapeutic interventions, may help more people experience healthy aging.

## 4 EXPERIMENTAL PROCEDURES

### 4.1 Cohort and covariate information

The Airwave Health Monitoring Study is an occupational cohort of employees of 28 police forces from across Great Britain. Full details of the cohort, methods and data access are available (Elliott et al., 2014). The study started recruitment in 2006 and now contains 53,280 participants. The study received ethical approval from the National Health Service Multi‐Site Research Ethics Committee (MREC/13/NW/0588). At the baseline health screening, participants underwent health examination, self‐completed a computer questionnaire and provided urine and blood samples. Blood samples were spun at the health clinic, and the biological samples were stored in a Thermoporter (LaminarMedica) and sent overnight from the clinics for next‐day analysis of standard clinical chemistry tests or were frozen at −80°C for long‐term storage. DNA samples and plasma for metabolomic analysis were extracted from blood collected in EDTA tubes.

Important covariates in the analysis were categorized from self‐report or clinical data as follows: ethnicity was defined as “white” or otherwise. Marital status was defined as living with partner or otherwise. Income was defined as low, medium or high, based on thirds of total net household income after adjustment for the number of dependant household members. Alcohol use was classed as nondrinker, moderate drinker (≤14 alcohol units/week for women and ≤21 alcohol units/week for men) or heavy drinker (> 14 alcohol units/week for women and >21 alcohol units/week for men). Hypertension was defined as ether reported diagnosis or systolic blood pressure ≥ 140 mmHg or diastolic blood pressure ≥ 90 mmHg. Diabetic status was defined as nondiabetic (no diagnosis and HbA1c < 6.5%), or diabetic (diagnosis or HbA1c ≥ 6.5%). Physical activity was defined as low, moderate or high based on the scoring protocol of the International Physical Activity Questionnaire (The IPAQ Group, 2016). Weekly reported average consumption of fruit, vegetables, red meat, and fish from a food‐frequency questionnaire was categorized into thirds, as low, medium and high consumption.

### 4.2 Psychological instruments

The Patient Health Questionnaire—9 depression questionnaire was used to define participants as “normal (i.e., no depression),” “minimal symptoms of depression” or as a “depression case” (Kroenke, Spitzer, & Williams, 2001). The Hospital Anxiety and Depression Scale questionnaire was used to assess anxiety levels as “normal (i.e., no anxiety),” “borderline” and “anxiety case” (Zigmond & Snaith, 1983). Participants were asked if they had experienced a work‐related traumatic incident in the previous six months. Those who reported a traumatic incident were then asked to complete a brief screening instrument for post‐traumatic stress disorder (PTSD) (Brewin et al., 2002). Participants were thus classed into three categories: “not experienced traumatic incident in past 6 months,” “experienced traumatic incident in past 6 months without leading to PTSD” and “experienced traumatic incident in past 6 months leading to PTSD”.

### 4.3 Metabolomic data acquisition

Metabolomic analysis of serum and urine was performed at the National Phenome Centre, based at Imperial College London. Samples were randomly sorted and thawed to 4°C, centrifuged to remove particulate matter, and the supernatant dispensed across dedicated 96‐well plates for each assay. Study reference (SR) samples, a pool of all samples for each matrix in the study, and long‐term reference (LTR) samples, a pool of samples external to study, were included in each analytical run to allow for quantification and correction of technical variation. Samples were maintained at 4°C during preparation for, and while awaiting, acquisition. Metabolomic data was acquired from urine using nuclear magnetic resonance spectroscopy (NMR, NOESY experiment) and ultra‐performance liquid chromatography–mass spectrometry (UPLC‐MS, hydrophilic interaction chromatography and reversed‐phase chromatography in both positive and negative modes) and from serum using NMR (CPMG experiment and lipoprotein subclass analysis) and UPLC‐MS (hydrophilic interaction chromatography and lipid‐targeted reversed‐phase chromatography in both positive and negative modes) (Table 1). Full details of metabolomic data acquisition and preprocessing, including processes to de‐noise the data for technical variation such as batch and run‐order, are given in the Supporting Information.

### 4.4 Metabolomic age modelling

Untargeted NMR data sets were glog‐transformed (Parsons, Ludwig, Gunther, & Viant, 2007), the quantified BiLISA data were log‐transformed, and the UPLC‐MS data were log‐transformed, following unit addition to every value to allow transformation of zero values. Data were then mean centred and scaled to unit variance.

Predictive models of age were constructed using elastic net regression (Zou & Hastie, 2005) in the “glmnet” package (Friedman, Hastie, & Tibshirani, 2010) in R ver. 3.3.2. using a multi‐step process. Elastic net model parameters, α (that defines mixing between lasso and ridge penalties) and λ (overall strength of penalty), were found following 10‐fold cross‐validation. A line search across α, between 0 and 1 in 0.01 increments, was performed to find the minimum mean cross‐validated error (*MSE*) using the optimal value of λ found using the “cvfit” command for each α value. The following steps were bootstrapped 100 times, re‐sampling the selected metabolomic data sets into different independent training (80%) and tests set (20%). *Step 1 Stability analysis:* Elastic net regression models were fitted on 100 further subsamples of the training data set (a random subsample of 80% each time). The metabolic features selected in each model were stored for each iteration. *Step 2 Metabolomic data restriction:* On the same subsample for 101 iterations, the number of metabolic features available to build an elastic net model was restricted by the percentage of iterations in step 1 that a feature was selected, moving from 100% to 0%, in 1% decrements for each subsequent iteration. The correlation between predicted and chronological age in remaining subsampled 20% of training set was stored for each iteration and the percentage restriction value that gave the best correlation was chosen for the final metabolic feature restriction in step 3. *Step 3 Final model building*: On the complete training data set, a final elastic net model was constructed using metabolic features restricted to those present in a set percentage of models, as found in step 2. *Step 4 Validation:* We assessed performance of models through Pearson's correlation (*r*) and mean absolute error (MAE) in the testing sets for each bootstrap.

To assess performance of each metabolomic platform, we first modelled age using each platform separately.

To define a metabolomic age combining platforms, we tested model performance using different platform combinations to improve practicality by using fewer metabolomic platforms since there is potential redundancy between metabolomic data sets (the same metabolite measured in multiple platforms) and because the different data structures of the various platforms may influence the variable selection process. We performed a leave‐platform‐out analysis by repeating the modelling process on a combined data set with one metabolomic platform left out each time. Platforms were removed from further analysis if model performed better (lower *MSE* calculated from 10‐fold cross‐validation) with their exclusion. We continued this process leaving further platforms out each time until no improvement in *MSE* was observed.

Metabolomic age was assigned to each participant based on the average predicted age from across all 100 bootstrapped models based on the final included platforms. Metabolomic age acceleration (mAA) was then defined as the difference between chronological age and metabolomic age, adjusted on chronological age as previously defined for DNA methylation age acceleration (Horvath, 2013). That is, we define mAA as the residuals of a linear regression between the chronological age and metabolomic age difference, with chronological age itself.

In sensitivity analyses, we repeat the modelling processes (on the final included platforms) using participants from different study centres, representing different regional police services, as the testing sets instead of bootstrapped sampling.

We also repeated the bootstrapping processing forcing BMI into each model. mAA was then defined in this analysis as the difference between chronological age and metabolomic age, adjusted on both chronological age and BMI.

### 4.5 Metabolic feature and pathway annotation

Tentative annotations were provided for mass spectrometry‐based metabolic features based on m/z searches across the Human metabolome database (Wishart et al., 2018), for the ion forms M+2H, M+H+NH4, M+NH4, M+H, M+ACN+H, M+CH3OH+H, M+Na, M+K, 2M+H at ±8 ppm mass tolerance.

For five UPLC‐MS‐based metabolic features that were both tentatively annotated by exact mass within our metabolomic age model and also available in repeat measurements within the Northern Finnish Birth Cohort data set, we performed further annotation procedures. Two of these annotations, for citrate (as in‐source fragmentation product) and leucine (M+Na ionic form), were supported by matching retention times and accurate mass to an internal reference standard database.

Significantly enriched metabolic pathways were predicted using the *Mummichog* program ver. 1.1.0 (Li et al., 2013), through the MetaboAnalyst platform (Chong et al., 2018). The enrichment analysis compared the sLPOS‐, uHPOS‐ and uRPOS‐derived features that were present in ≥75% of models (the feature list), to features present in these data sets but not selected into ≥75% of models (the reference list). The algorithm searches tentative compound lists from metabolite reference databases against an integrated model of human metabolism to identify functional activity. Fisher's exact tests are used to infer p‐values, which are adjusted for type I error through a pathway permutation procedure and for likelihood of pathway enrichment among significant features as compared to pathways identified among the entire compound set present in reference list (the entire metabolome data set), considering the probability of mapping the significant m/z features to pathways. Mummichog parameters were set to match against ions included in the “positive mode” setting at ±5 ppm mass tolerance. Visualization of enriched pathways on the KEGGscape network was performed through the MetaboAnalyst platform (Chong et al., 2018).

### 4.6 Metabolite validation in the Northern Finnish Birth Cohort 1966

The Northern Finnish Birth Cohort 1966 is a prospective birth cohort that sampled 12,058 live births in 1966, including 96.3% of all births in the regions of Oulu and Lapland in Finland (Rantakallio, 1988). Fasting blood samples were collected at follow‐up of participants at ages 31 and 46 years and stored at −80°C for subsequent biomarker profiling. A high‐throughput NMR metabolomics platform was used for the analysis of 149 metabolic measures (Soininen, Kangas, Wurtz, Suna, & Ala‐Korpela, 2015). This metabolomics platform provides simultaneous quantification of routine lipids and lipid concentrations of 14 lipoprotein subclasses and major sub‐fractions, and further quantifies abundant fatty acids, amino acids, ketone bodies and gluconeogenesis‐related metabolites in absolute concentration units.

We assessed changes of 11 metabolites, that were available in this data set and also included in our predictive model, between these two sampling points using 1‐tailed *t* tests. We used a validation *p*‐value of .005 (.05/11 tests) to infer statistical significance.

### 4.7 DNA methylation analysis

Full details of DNA methylation data acquisition and preprocessing are given in the Supporting Information.

DNA methylation age was computed according to the algorithm described by Hannum et al. (2013) based on 71 blood‐specific CpG sites and the algorithm of Horvath (2013) based on 353 nontissue‐specific CpG sites. DNA methylation phenotypic age was computed according to the algorithm of Levine et al, based on 513 CpG sites (Levine et al., 2018). Age acceleration (AA) was defined as the difference between epigenetic and chronological age. Since AA could be correlated with chronological age and WBC percentage, we computed the so‐called intrinsic epigenetic age acceleration for Horvath and Hannum age (Chen et al., 2016), which is defined as the residuals from the linear regression of AA with chronological age and blood cell counts (measured using flow cytometry) for neutrophils, lymphocytes, monocytes and eosinophils. Phenotypic age acceleration was defined as residuals from the linear regression of AA with chronological age (Levine et al., 2018).

### 4.8 Analysis of risk factors of biological age acceleration

We analysed associations between mortality risk factors and age acceleration scores in separate adjusted linear regression models, using age acceleration scores as the dependent variables and the risk factor as the independent variable. To allow comparison across risk factors and age scores, the adjustment set, included in all models, was chosen a priori*.* It included demographic variables (sex, ethnicity, study centre, income), the 25 × 25 main NCD risk factors, (hypertension, diabetes, BMI, smoking, alcohol intake, physical activity) and dietary indicators (red meat, fish, fruit and vegetable consumption). We corrected for assessment of multiple exposures using the 5% false discovery rate (Benjamini & Hochberg, 1995).

## CONFLICT OF INTEREST

All authors have no conflicts of interest to declare.

## AUTHOR CONTRIBUTIONS

OR and PV conceived the study. OR performed most analyses and drafted manuscript. EH contributed additional analyses and ML supervised metabolomic data acquisition. RCP processed the mass spectrometry data. IK processed the NMR data. IT supervised metabolomic data curation. AH, HG and RP prepared and supervised data collection in AIRWAVE cohort. MRJ and MW prepared and supervised data collection in NFBC cohort. MAK was responsible for the NMR metabolomics in the NFBC cohort. GF and SP acquired and processed DNA methylation data. PE coordinated and supervised AIRWAVE cohort. All authors critically reviewed the manuscript.

## Supporting information

Supplementary Material

Supplementary Material

## Data Availability

All AIRWAVE metabolomic data sets are available for download here https://doi.org/10.14469/hpc/6945. DNA methylation data are available for download from the Gene Expression Omnibus (GEO) repository at https://www.ncbi.nlm.nih.gov/geo/query/acc.cgi?acc=GSE147740. All other AIRWAVE data may be accessed upon application to Dementias Platform UK Data portal (https://portal.dementiasplatform.uk/Apply/ApplicationProcess). Application to obtain Northern Finnish Birth Cohort metabolomic data should be made to https://www.oulu.fi/nfbc/node/47960.
